# Psychological Impact of the Lockdown in Italy Due to the COVID-19 Outbreak: Are There Gender Differences?

**DOI:** 10.3389/fpsyg.2021.567470

**Published:** 2021-03-16

**Authors:** Nadia Rania, Ilaria Coppola

**Affiliations:** Department of Education Sciences, University of Genoa, Genoa, Italy

**Keywords:** COVID-19, happiness, psychological well-being, loneliness, mental health, gender differences, Italy

## Abstract

The COVID-19 emergency has hit the whole world, finding all countries unprepared to face it. The first studies focused on the medical aspects, neglecting the psychological dimension of the populations that were forced to face changes in everyday life and in some cases to stay forcedly at home in order to reduce contagion. The present research was carried out in Italy, one of the countries hardest hit by the pandemic. The aim was to analyze the perception of happiness, mental health, and the sense of loneliness experienced by adults during the lockdown due to the COVID pandemic. Specifically, the variables will be examined in relation to gender difference, living alone, with partner, or with partner and children. The research followed a quantitative approach using an online questionnaire. The project involved 1100 subjects from whom 721 participants (75.5% women) were extrapolated. Of them, 17.3% claimed to live alone, 39.5% with their partner, and 43.1% with their partner and children. The results show that people in general experienced a lower level of happiness and mental health and higher levels of loneliness compared to normative sample. The lockdown and pandemic condition due to COVID-19 seems to have canceled the gender differences in the perception of happiness and mental health, while it seems to have increased the perception of loneliness experienced by males compared to the pre-pandemic condition. In addition, those who lived alone perceived a greater level of loneliness than those who lived with their partner or partner and children. Unexpectedly, no significant differences emerged regarding the level of happiness and mental health between those who had direct contact with the virus and those who did not. These data should make political decision-makers reflect on the need to pay more attention to the implications that such drastic measures as a lockdown can have on people’s psychological well-being.

## Introduction

On January 30, 2020, the World Health Organization declared the COVID-19 epidemic a public health emergency of international interest (World Health Organization, 2020a), although this information was not disclosed by the media in the various countries but only after the situation manifested itself in Europe with countless deaths. Many people stayed at home and socially isolated themselves to prevent being infected, both in China and in other countries or because it was imposed by governments or because individuals considered it the only way to protect themselves. The first signs of the COVID-19 in Italy dated to January 31, 2020, but the first outbreak of infections was detected on February 21, 2020, followed by the first deaths. As a result of the first outbreaks, some municipalities were immediately quarantined and subsequently restrictive measures, progressively more stringent, were extended to the entire country, not allowing the population to leave their homes if not strictly necessary and blocking all activities not related to food production. Despite these measures, Italy appears to be the third country in the world for number of positive cases and the second in the world for number of deaths (European Centre for Disease Prevention and Control, 2020). In this scenario, the management of the coronavirus epidemic (COVID-19) by the Italian government, with highly restrictive measures compared to other countries, has made it impossible for the population to continue leading a normal life. Alongside the very strong media attention linked to physical and behavioral restrictions, the government failed to consider the psychological and social aspects that such decisions could have on the population. The government task force also focused on the medical and health aspects as did the international and national scientific literature in the first articles published in the early months of 2020 on the COVID-19 pandemic (COVID Contents n. 1 of 9 April 2020; Istituto Superiore di Sanità [ISS], 2020a, COVID Contents n. 2 of 16 April, 2020, Istituto Superiore di Sanità [ISS], 2020b Study Group COVID-19, Contents n. 1 of 9 April, 2020).

### Mental Health and COVID-19

Mental well-being has been conceptualized in various ways highlighting the multidimensionality of the concept. Diener et al. (1999) depicted subjective well-being as consisting of cognitive (judgment about one’s life satisfaction) and affective (balance between positive and negative emotions) aspects. Seligman (2011), on the other hand, introduced a model where psychological well-being encompassed the following domains: positive emotions, engagement, relationships, meaning, and accomplishment. Furthermore, several definitions of mental health have been given over the years: Galderisi et al. (2015) underline how first it was described only as the absence of mental illness and later as a state of well-being that allows the individual to cope with stressful life events, to carry out work in a productive way, and to make a contribution to their own community (World Health Organization, 2004). However, the authors, underlining the possible misunderstandings that could have arisen from this definition, proposed a further one: “Mental health is a dynamic state of internal equilibrium which enables individuals to use their abilities in harmony with universal values of society. Basic cognitive and social skills; ability to recognize, express, and modulate one’s own emotions, as well as empathize with others; flexibility and ability to cope with adverse life events and function in social roles; and harmonious relationship between body and mind represent important components of mental health which contribute, to varying degrees, to the state of internal equilibrium” (Galderisi et al., 2015, pp. 231–232). Other authors argue that mental health is an ambiguous concept (Van Droogenbroeck et al., 2018), one of the basic components in the broader dimension of an individual’s general health and therefore difficult to define (Kvrgic et al., 2013). Mental health, in fact, is part integral to health and well-being and can be influenced by several interacting psycho-social, biological, and demographic factors (such as sex, age, and family environment; World Health Organization, 2001; Kvrgic et al., 2013).

Focusing on mental health is of fundamental importance when entire nations are facing catastrophic events, such as the recent one caused by COVID-19, which can compromise the mental health of citizens. However, it is not the first time that entire nations have faced a catastrophic situation due to an uncontrollable medical condition, with repercussions on the economic, political, social, and individual systems. In fact, think of the 2003 epidemic caused by Severe Acute Respiratory Syndrome (SARS) or even the most recent swine flu of 2009. In these cases, the literature had mainly focused on the psychological implications suffered by the patients directly involved and by the medical and healthcare personnel working on the front line: the emotional reactions experienced by those who during the SARS epidemic worked closely with the disease were extremely intense and included fear of contagion, feelings of stigma, loneliness, boredom, anger, anxiety, stress, and a sense of uncertainty (Maunder et al., 2003; Al-Rabiaah et al., 2020). Chua et al. (2004) also found that during the 2003 outbreak, stress levels were high in both patients and healthy participants, indicating that the whole community had been affected regardless of the educational level. Patients also reported feelings of loneliness and boredom caused by prolonged quarantine (Chua et al., 2004) and a high prevalence of psychological distress (Hawryluck et al., 2004).

In relation to the COVID-19 pandemic, most of the research initially focused mainly on the medical aspects, in particular on identifying the epidemiology and clinical characteristics of infected patients (Chen, 2020), on the genomic characterization of the virus (Lu, 2020), on comorbidity with other diseases such as cardiovascular and diabetes mellitus (Kuno et al., 2020), and on the global health challenges (Rubin and Wessely, 2020). Other areas of investigation have focused on the pathology’s symptoms associated with acute respiratory distress syndrome (Xu et al., 2020) or the symptoms of posttraumatic stress disorder in patients with stable COVID-19, highlighting how many of these patients suffer from significant PTSD symptoms at the time of discharge with possible negative consequences on their quality of life and work performance.

These data highlight the need for appropriate psychological interventions especially for stabilized COVID-19 patients (Duan and Zhu, 2020). In this regard, online mental health services have been organized in the Chinese context to intercept the psychological needs of the population, who had COVID-19 but also in general, in order to improve the quality and effectiveness of the emergency interventions (Liu S. et al., 2020).

Furthermore, particular attention should be paid to the psychological problems of those who work not only in the front line in the COVID-19 emergency, for example, medical personnel and nurses but also to the population in general, given that vicar traumatization scores of the general population were found to be significantly higher than those of nurses on the front line (Li et al., 2020).

During the COVID-19 pandemic, the attention was also focused on the psychological repercussions that this situation could cause in healthcare workers especially in stress management (Greenberg et al., 2020; Joob and Wiwanitkit, 2020) caused by the work overload, frustration, and being faced with very difficult choices on a daily basis. It is therefore essential to take into consideration the psychological health of healthcare professionals to prevent the onset of possible issues not only during the critical stages of the epidemic but also in the following ones, to prevent long-term consequences. The mental health of health workers is indeed essential to better controlling infectious diseases and better responding to future unexpected infectious diseases (Chen et al., 2020). As highlighted by Zowalaty and Järhult (2020), the success in containing the COVID-19 pandemic will depend on the ability of the various countries to adopt public health measures capable of identifying clinical cases, to implement a rigorous control of infections in healthcare facilities, to isolate patients, and to be able to contain the spread of the virus in the community and in public education contexts.

Still few research studies have examined the psychological impact of COVID-19 on the general population within the first weeks of the COVID-19 outbreak and related lockdown. The first data have shown symptoms of anxiety, sleep disorders, depression, lower mental well-being, and psychological distress in the general population and the importance of monitoring these dimensions (Ahmed et al., 2020; Brooks et al., 2020; Casagrande et al., 2020; Ko et al., 2020; Moccia et al., 2020; Tian et al., 2020; Wang C. et al., 2020; Wang Y. et al., 2020; Yang and Ma, 2020). Ahmed et al. (2020) also highlighted how the confinement in their homes, due to the COVID-19 epidemic, led to higher levels of anxiety, depression, and a lower level of mental well-being. In Italy, attention has been focused in particular on specific groups such as university students (Capone et al., 2020) and families (Centro di Ateneo Studi e Ricerche sulla Famiglia, Università Cattolica del Sacro Cuore, 2020; Ferrario and Profeta, 2020; Lagomarsino et al., 2020; Libellula Foundation, 2020), and in a few cases on the general population (Ferrucci et al., 2020; Pakenham et al., 2020).

### Emotions and Mental Health

Emotions are fundamental components of the life of human beings from which people draw the stimuli that activate their daily activities. However, even if every emotion is important, the search for positive sensations such as happiness affords an emotional state of well-being and a general realization. The research on happiness conducted in recent years (Luhmann et al., 2012) follows two approaches: studies on eudaimonic well-being, which focuses on psychological well-being, and those on hedonic well-being, which focus on subjective well-being (Ryff and Keyes, 1995). According to Diener et al. (1999), happiness can be considered a dimension of the complex and multidimensional concept of psychological well-being. Among the different notions of happiness presented in the literature, Lyubomirsky et al. (2005) define it as the shortest way to refer to the experiences of frequent positive emotions. Their review also shows how happiness is positively correlated with health indicators, both mental and physical; this could be due to the fact that it has effects on social relationships, on coping skills (Lyubomirsky et al., 2005; Piqueras et al., 2011), and on stress (Lyubomirsky et al., 2005; Heizomi et al., 2015). Furthermore, happiness and depression are considered two dimensions of the complex and multidimensional concept of well-being (Diener et al., 1999).

Considering emotional well-being during quarantine can be important for understanding the impact that this situation has on the general well-being of people. The ongoing COVID-19 epidemic is generating negative emotions like fear as already argued by Khalid et al. (2016), above all with regard to the emotions of healthcare workers during a Middle East Respiratory Syndrome-Coronavirus outbreak, but also recently underlined by Horton (2020), who proposed “A desperate plea from an ordinary citizen in China.” Furthermore, both studies on previous and current pandemics have highlighted how the epidemic situation has a negative impact on the dimension of happiness (Yip et al., 2010; Yang and Ma, 2020). As pointed out by Wang C. et al. (2020), previous research has revealed multiple psychosocial impacts both on an individual level such as fear of getting sick or dying (Hall and Chapman, 2008) or the negative emotions experienced by individuals following the closure of schools and businesses (Van Bortel, 2016), but also the psychological impacts on the uninfected population, revealing significant psychiatric morbidity (Sim et al., 2010). Furthermore, Cipolletta and Ortu (2020) underline how COVID-19 emergency suspended time, causing uncertainty and anxiety, both for the future and for the coronavirus (“coronaphobia”; Asmundson and Taylor, 2020), which can be reduced making meaning to the events. Moreover, Yang and Ma (2020) found how the outbreak of the epidemic influenced people’s emotional well-being, identifying some factors that can further affect it, such as the probability of contracting a disease and developing relationship problems, while the perception of greater knowledge of the epidemic increases the sense of control, thus becoming a protective factor for emotional well-being.

### Loneliness and Mental Health

Among the relevant risk factors of depression, there is the dimension of loneliness; therefore, happiness and loneliness can be considered two components of individual subjective well-being capable of defining depressive risk conditions (Cacioppo et al., 2006) as well as mental health. In the literature, several authors have been interested in the concept of loneliness, and Henriksen et al. (2019) considered loneliness a very common condition in Western communities. In the past, it has been defined as a complex set of feelings that include reactions to the absence of intimate and social needs (Ernst and Cacioppo, 1999), a problem for society (Cacioppo and Cacioppo, 2018) as well as for the individual, which may have serious health consequences (Luanaigh and Lawlor, 2008; Henriksen et al., 2019). Hyland et al. (2019) argue that loneliness has traditionally been presented as a one-dimensional concept, when in reality it is a multidimensional construct (Cacioppo et al., 2015; Hyland et al., 2019). In particular, some authors (Hawkley et al., 2005, 2012; Cacioppo et al., 2015) present loneliness as a construct that includes three connected dimensions: intimate loneliness, relational loneliness, and collective loneliness. Hyland et al. (2019), instead, present loneliness as a construct defined by four quantitatively and qualitatively differentiated classes: low, social, emotional, and “social and emotional.” Their study also revealed that the perceived quality, rather than quantity, of interpersonal connections was associated with poor mental health. In fact, as pointed out by Cacioppo and Patrick (2008) and Cacioppo et al. (2015), the construct of loneliness clearly highlights how the human species needs significant others, people to trust, and with whom to plan life. Finally, some authors have found that loneliness is a risk factor for the development of depressive symptoms and is negatively associated with life satisfaction and positive affect (Cacioppo et al., 2010; Chang et al., 2020). It follows that exploring these constructs, still little investigated in the literature on pandemics, is very important. Some authors have found (Porcelli, 2020; Tull et al., 2020) that compulsion at home during the COVID-19 lockdown was associated with greater stress, social isolation, loneliness, and anxiety about their health and the economic aspects.

### Gender Differences During the COVID-19 Pandemic

In relation to mental health, the literature confirms sharp differences between the genders. It is a constant in the literature that women reported worse mental health outcomes (Kvrgic et al., 2013; Giorgi et al., 2014; Van Droogenbroeck et al., 2018). Even during the period of the COVID-19 pandemic, the first data collected by the researchers highlight this trend (Casagrande et al., 2020; Wang C. et al., 2020; Wang Y. et al., 2020).

Furthermore, from the first studies on the psychological effects due to COVID-19, it was found that women were more affected by this situation, manifesting more negative alterations in cognition or mood and hyperarousal than males (Liu N. et al., 2020).

Moreover, also with regard to loneliness, there are gender differences in the literature. Some studies show a higher prevalence of loneliness among women (Pinquart and Sorensen, 2003; Henriksen et al., 2019). Hyland et al. (2019) emphasize that males, mirroring women, show higher social loneliness and less emotional loneliness. In fact, women tended to fall more into the emotional loneliness class, while no differences emerged in relation to “social loneliness.”

Research has shown that, in particular, during the COVID-19 epidemic, women were most exposed to stress and depressive symptoms (Casagrande et al., 2020; Wang C. et al., 2020; Wang Y. et al., 2020), while, in some cases, being men has been associated with to be a protective factor against the risk of mild psychological distress in response to stressful events (Moccia et al., 2020). Even a month after the first cases, it was found that women showed symptoms of posttraumatic stress disorder, to a greater extent than men, in terms of the negative alternation of mood and cognition and hyperarousal (Liu N. et al., 2020). Unlike other studies, Ahmed et al. (2020), despite having identified higher levels of anxiety and depression and a lower level of mental well-being in the population, did not, however, find gender-related differences.

Therefore, in the light of this situation, a timely understanding of mental health status, an aspect surprisingly overlooked, is urgently needed for society. As can be seen from the analysis of the current literature, little is known about the psychological impact, mental health and well-being of the general population, and the related gender differences at the peak of the COVID-19 epidemic worldwide, while this is one of the first Italian studies to have focused on the population in general to understand the effects of the lockdown.

## Aims of the Current Study

Considering this theoretical and contextual framework, the present study aims to investigate psychological well-being in relation to some social dimensions during the initial quarantine period that the Italian population had to cope with. In particular, the research objective is to analyze the perception of happiness, mental health, and the sense of loneliness experienced by the adult Italian population during this period. Specifically, the variables will be examined in relation to gender differences, living alone, living with partner, living with partner and children, or having family members or friends who had COVID-19. Furthermore, we want to investigate the relationships between the variables considered and the dimensions that affect well-being and loneliness.

## Materials and Methods

This study is part of a larger multidisciplinary, anonymous online survey; only some specific variables concerning the psychological field and related to the proposed objectives will be presented here. The research design is longitudinal, and in this paper, present data relating to first lockdown in Italy started on March 11th, 2020, with Dpcm #IoRestoaCasa (#IStayatHome). The survey was carried out over a 10-day period, after the first 2 weeks of lockdown, from March 25th to April 4th, 2020. This situation forced most of the population not to work or to engage in smart working and students not to attend face-to-face lessons and to follow lessons via distance learning. The methodology used is quantitative, and the protocol is based on previous studies on the psychological impacts of SARS and influenza outbreaks (Rubin et al., 2010; Park et al., 2018; Al-Rabiaah et al., 2020), as well as on the few studies that had already been published in the psychological field relating to the COVID-19 pandemic in early March 2020 (Liu S. et al., 2020; Rajkumar, 2020; Wang C. et al., 2020). Furthermore, general health, as seen from the analysis of the literature, is a composite construct and, as several authors point out happiness and loneliness are two components of individual subjective well-being. Therefore, taking into account these considerations, in order to measure psychological well-being, a protocol was prepared that included the following scales widely used in the literature and specific questions related to the COVID-19 outbreak.

### Measures

• *Subjective Happiness Scale (SHS)* measures the subjective global happiness, developed by Lyubomirsky and Lepper (1999), and in this study, we used the Italian version by Iani et al. (2013). The scale is made up of four items (e.g., “*Some people are generally not happy. They enjoy life regardless of what happens and take the best of everything. How much does this phrase describe it*?”) with a Likert response scale, from 1 = *not at all* to 7 = *very much*. The total score ranges from 4 to 28 points; higher scores indicate higher levels of happiness. The scale showed a good internal consistency (α = 0.58).
• *General Health Questionnaire* (GHQ-12; Goldberg and Williams, 1988; Italian version Piccinelli and Politi, 1993) consists of 12 items that assess the severity of a mental problem over the past few weeks. Participants have to report whether they have experienced a particular symptom of mental distress on a four-point Likert-type scale from 0 = *less than usual* to 3 = *much more than usual*. The six positive items were corrected from 0 (much more than usual) to 3 (less than usual) and the six negative ones from 3 (much more than usual) to 0 (less than usual). The total score ranges from 0 to 36 points; higher scores indicate worse health. The scale showed a good internal consistency (α = 0.83). GHQ-12 is widely used for mental health trend analysis for its ease of use, breadth of distribution, and capacity to reproduce “remarkably robust” results contrasted with longer initial versions (Griffith and Jones, 2019).
• *Loneliness Scale*: we used the Three-Item Loneliness Scale developed by Hughes et al. (2004) from the revised UCLA Loneliness Scale (Russell et al., 1980), Italian version of the revised UCLA Loneliness Scale (Solano and Coda, 1994). It is a short scale for measuring loneliness in large surveys, and it assesses feelings of isolation, disconnectedness, and not belonging. Respondents are placed on a three-point Likert scale from 1 = *hardly ever* to 3 = *often*, with a total score ranging from 3 to 9 points; higher scores indicate greater loneliness. The three-item scale showed a good internal consistency (α = 0.60).
• Compilation of a socio-demographic data sheet which included age, gender, education, type of work during the COVID-19 health emergency, size of the home, income, with whom the subject lives during the COVID-19 health emergency, and having family members or friends who had COVID-19.

### Procedure

The online study was promoted through email, WhatsApp, discussion forums, and social networks such as Facebook. The call to the study, with indications of the purpose of the study, the tools proposed, and the type of restitution, included a link to access the questionnaire. Before filling out the questionnaire, subjects had to read the informed consent, declare to agree, to be of age, to have understood that participation was voluntary, and that they could withdraw at any time by closing the browser window. The convenience sample was recruited through random cascade sampling, starting from some subjects known by the research team. The research, therefore, is characterized by its exploratory nature which does not aim to return a representative image of the Italian population but propose a picture of the perceptions of the population during the lockdown in relation to their psychological well-being. It took an average about of 22 min for each participant to fill out the survey.

The data were collected in compliance with the privacy and research ethics code of the Italian Association of Psychology, after the protocol was approved by the Ethics Committee of the Department of Education Sciences of the University of Genoa.

### Data Analysis

Descriptive statistics were calculated for sociodemographic characteristics and information about variables, and the SHS, GHQ, and UCLA scores were expressed as mean and standard deviation. To investigate the gender differences in relation to SHS, GHQ-12, and UCLA, *t*-tests were used for independent samples. Also, to investigate differences between those who had or did not have family members or friends with COVID-19 in relation to SHS, GHQ-12, and UCLA, *t*-tests were used for independent samples. To compare the differences between our participants and the Italian normative sample for SHS (Iani et al., 2013), GHQ (Giorgi et al., 2014), and UCLA (Caputo, 2017), *t*-tests were conducted for single samples. The Italian samples to which reference was made to perform *t*-tests were chosen on the basis of the socio-demographic characteristics of the participants (gender, age, and non-clinical sample), who took part in the research carried out prior to the COVID-19 [Iani et al. (2013) for SHS, Giorgi et al. (2014) for GHQ-12, and Caputo (2017) for UCLA]. The characteristics were similar to those possessed by the participants in our research. While ANOVA was used to investigate the differences between groups (I live alone, I live with my partner, and I live with my partner and children or people), with *post hoc* Tukey (for homogeneous variances) between group comparisons in case of a significant overall *F*-value. Appropriate effect size statistics that adjust for differences in group sizes were obtained of Cohen’s *d* for *t*-tests and η^2^*_*p*_* for ANOVAs.

To explore the relationship between the SHS, GHQ, and UCLA scales, correlation analyses were performed. We used multiple linear regressions to calculate the univariate associations between sociodemographic characteristics, and SHS, GHQ, and UCLA scales. Statistical analysis was performed using SPSS Statistic 18.0.

### Participants

Overall, the sample comprised 1100 participants, distributed throughout the national territory, from whom 721 subjects who claimed to live alone (17.3%), with partner (39.5%), or with partner and children (43.1%) were extrapolated. The majority of respondents were women (75.5%), with an average age of 49.48 years (SD = 12.71, range 22–81), while men had an average age of 52.32 years (SD = 12.85, range 26–83). In Table 1, socio-demographic variables of our sample are reported.

**TABLE 1:** Sociodemographic characteristics of the participants (*N* = 721).

**Category variables**	**%**
*Gender*	
Male	24,5
Female	75,5
*Marital status*	
Unmarried	15.1
Married/cohabiting	74.3
Separate/divorced	8.2
Widower	2.4
*People with the subject lives*	
Alone	17.3
With partner	39.5
With partner and children	43.1
*Educational qualification*	
Junior high school	3,4
Secondary school	34.1
Graduation	42.6
Postgraduate specialization	20.0
*Work arrangements during COVID-19*	
Unchanged	24,5
Smart-working	57.0
Loss of job/work permit/leave	18.5
*Household income*	
Up to 15,000 euros	11.9
Between 15,001 and 28,000 euros	30.1
Between 28,001 and 55,000 euros	37.3
Between 55,001 and 75,000 euros	11.6
Over 75,000 euros	9.1
*Contact with COVID-19*	
Subjects that have had some contact with COVID-19	62.8
Subjects that have had COVID-19	6.7
Subjects that have had relatives or friends with COVID-19	27.2
Subjects that believe they or that relatives and friends had COVID-19	24.8
Subjects knew people close to them who did not survive COVID-19	19.8

Over half of the sample came into contact with COVID-19 (62.8%), of whom 6.7% had it directly, 27.2% had relatives and friends, 24.8% believed they had it, or that relatives and friends had it, but they were not sure because no swab tests were done, and 19.8% knew people close to them who did not survive COVID-19.

## Results

The descriptive statistics of each variable used to measure the psychological well-being impact are presented in Table 2. It included mean and standard deviation in relation to gender and the categorical variable “people with the subject lives.”

**TABLE 2:** Descriptive statistics of variables of the sample (*N* = 721).

**Sociodemographic variables**	**Subjective happiness SHS**		**Mental health GHQ-12**		**Loneliness UCLA**				
	***M***	**SD**	***M***	**SD**	***M***	**SD**	***t(df)/F***	***p***	**Cohen’s *d/η^2^_p_***
*Gender*							−2.73 (672)	0.007	0.24
Male	4.51	0.85	17.51	5.63	5.23	1.70			
Female	4.44	0.92	18.23	6.13	5.67	1.97			
*People with the subject lives*							3.83	0.022	0.011
Alone	4.42	0.85	17.29	6.24	5.98	2.19			
With partner	4.45	0.98	18.20	5.64	5.50	1.87			
With partner and children	4.47	0.91	18.12	6.21	5.43	1.85			

The SHS revealed a mean score above the theoretical average, with average scores practically equal between males and females. The respondents’ mental health levels, measured using the GHQ, revealed mean scores near the theoretical average for both males and females with slightly higher malaise scores for females. For the Loneliness scale (UCLA), participants had a mean score near the theoretical mean score.

Specific analyses on gender differences did not show statistically significant differences for either SHS or GHQ scale, while for UCLA scale, significant gender differences emerged, with women showing significantly higher mean scores (*M* = 5.67; DS = 1.97) than the males of the sample (*M* = 5.23; DS = 1.70) [*t*(319.9) = −2.73, *p* < 0.05, Cohen’s *d* = 0.24]. Furthermore, there were no significant differences in the variables considered between those who had direct contact with the COVID-19 (themselves, relatives, or friends) and those who did not.

Also, as regards living alone, with partner, and with partner and children, in all three scales considered, the scores were always close to the theoretical averages. Furthermore, no significant differences emerged in SHS and GHQ regarding the variable “*People with the subject lives*”; significant differences emerged instead for UCLA. In fact, there was a significant difference between those living alone and those living with partner or with partner and child in the level of loneliness, *F*(2,681) = 3.83, *p* < 0.05, and η^2^*_*p*_* = 0.011. *Post hoc* testing revealed a significant difference between those living with partner (*M* = 5.50, SD = 1.87) and who live with partner and children (*M* = 5.43, SD = 1.85) having a lower level of loneliness than those living alone (*M* = 5.98, SD = 2.11). These finding indicated that there was a higher level of loneliness among those living alone than those living with partner or with partner and children.

Moreover, comparing the SHS data of the participants with those obtained on the Italian validation scale (Iani et al., 2013), the analysis of the means for a single sample showed that our participants presented significantly lower average happiness scores, measured in relation to the COVID-19 emergency (Table 3).

**TABLE 3:** Subjective Happiness Scale (SHS) comparison between the average values of the participants and the average values of the Italian normative sample.

	**SHS**				
	**Participants**	**Italian normative sample**	***t* (df)**	***p***	**Cohen’s *d***
	***M* (SD)**	***M* (SD)**			
Male	4.51 (0.85)	4.74 (1.22)	−3,414 (166)	0.001	0.22
Female	4.44 (0.92)	4.80 (1.21)	−8,791 (500)	0.000	0.33

As regards the GHQ scale, respondents scored significantly higher than the Italian normative sample (Giorgi et al., 2014) indicating a “worse degree” of mental well-being (Table 4).

**TABLE 4:** Mental health comparison between the average values of the participants and the average values of the Italian normative sample.

	**GHQ-12**				
	**Participants**	**Italian normative sample**	***t* (df)**	***p***	**Cohen’s *d***
	***M* (SD)**	***M* (SD)**			
Male	17.51 (5.63)	9.8 (4.9)	17,633 (165)	0.000	1.47
Female	18.23 (6.13)	11.1 (5.7)	25,647 (485)	0.000	1.20

The scores on the UCLA scale (Table 5), on the other hand, compared with the Italian normative sample (Caputo, 2017) showed significant differences only in males, who obtained higher loneliness scores than the normative sample, while the females, though having slightly higher scores than the males, did not present differences with the normative sample considered.

**TABLE 5:** Loneliness Scale: comparison between the average values of the participants and the average values of the Italian normative sample.

	**UCLA**				
	**Participants**	**Italian normative sample**	***t* (df)**	***p***	**Cohen’s *d***
	***M* (SD)**	***M* (SD)**			
Male	5.23 (1.71)	4.94 (1.92)	2.002 (165)	0.047	0.14
Female	5.68 (1.97)	5.58 (2.08)		NS	

A next level of analysis was the correlations between the constructs considered in the study. All the constructs considered correlate with each other. There was a moderate positive correlation between GHQ scale and UCLA scale (*r* = 0.32, *p* = 0.01), while there was a moderate negative correlation between SHS and GHQ scale (*r* = −0.40, *p* = 0.01) and SHS and UCLA scale (*r* = −0.32, *p* = 0.01).

Based on the main correlations highlighted, further investigation highlighted the factors affecting mental health, happiness, and loneliness. The stepwise model selection in multiple linear regression analysis, which considered GHQ scale as a dependent variable, is presented in Table 6.

**TABLE 6:** Regression model: Mental Health (GHQ) as dependent variable.

**Variables**	***B***	**SE**	**Beta**	***t***	***R*^2^**
SHS	−2.110	0.354	−0.314	−5.968	0.195
UCLA	0.640	0.162	0.208	3.955	
Home	−1.409	0.607	−0.116	−2.321	

The model had an *R*^2^ = 0.20, which means that 20% of the variance in the GHQ scale is explained by the model. The *R*^2^ value was statistically significant. SHS seems to be the biggest predictor (β = −0.31, *p* < 0.001), while UCLA (β = 0.21, *p* < 0.001) and the size of the home (β = 0.12, *p* < 0.05) were moderate predictors.

Table 7 presents the stepwise model selection in multiple linear regression analysis, in which SHS was used as a dependent variable.

**TABLE 7:** Regression model: Happiness (SHS) as dependent variable.

**Variables**	***B***	**SE**	**Beta**	***t***	***R*^2^**
GHQ	−0.054	0.007	−0.361	−8.317	0.228
UCLA	−0.107	0.021	−0.225	−5.178	

The model had an *R*^2^ = 0.23, which means that 23% of the variance in SHS is explained by the model. The *R*^2^ value was statistically significant. The GHQ scale seemed to be the biggest predictor (β = −0.36, *p* < 0.001), while UCLA scale (β = −0.23, *p* < 0.001) seemed to be moderate predictors.

Table 8 presents the stepwise model selection in multiple linear regression analysis, in which the UCLA scale was used as a dependent variable.

**TABLE 8:** Regression model: Loneliness (UCLA) as dependent variable.

**Variables**	***B***	**SE**	**Beta**	***t***	***R*^2^**
SHS	−0.497	0.121	−0.228	−4.125	0.162
GHQ	0.076	0.018	0.233	4.214	
People with the subject lives	−0.186	0.074	−0.128	−2.513	
Educational	−0.284	0.132	−0.110	−2.160	

The model had an *R*^2^ = 0.16, which means that 16% of the variance in the UCLA scale is explained by the model. The *R*^2^ value was statistically significant. SHS seemed to be the biggest predictor (β = −0.23, *p* < 0.001), while GHQ scale (β = 0.23, *p* < 0.001) with People with the subject lives (β = −0.13, *p* < 0.05), and the qualification (β = −0.11, *p* < 0.05) were moderate predictors.

## Discussion

Italy has been hit by a sudden traumatic situation linked to the COVID-19 pandemic that has led the population to a forced lockdown. This situation, as shown by the data of the present study, had a significant psychological impact on its inhabitants.

The data underline how this event has canceled gender differences in the perception of happiness (SHS) and mental health (GHQ-12). In these variables, women usually have a significantly lower score than men. The data, however, show that men and women had similar and significantly lower scores than the pre-pandemic condition; as far as subjective happiness is concerned, the emotional well-being (Iani et al., 2013) scores were significantly higher than mental health (Giorgi et al., 2014), highlighting a worsening of the mental psychological component. These data are also confirmed by recent literature which highlights how the epidemic that has hit the whole world has a negative impact on the perception of happiness (Yang and Ma, 2020) and on mental health in general (Ahmed et al., 2020). Unlike what could be expected, however, no significant differences emerged regarding the level of happiness and mental health between those who had direct contact with the virus and those who did not have it, as if the pandemic condition was so pervasive as to make everyone more vulnerable regardless of whether they are personally affected or not. As regards, however, the data relating to the dimension of loneliness showed significantly higher scores for women than for males. Although comparing our data with the normative sample, significant differences emerge only for males who scored significantly higher than the pre-pandemic condition, as if compulsion at home had increased the perception of loneliness more in men than in women. In practice, the perception of loneliness of women that is normally higher than men did not change during the pandemic compared to the pre-pandemic condition. The male gender therefore seems to have worsened this dimension by perceiving more the dimension of loneliness due to forced isolation at home. These data could be interpreted by the fact that men are probably less used to spending time in the house as opposed to women who are more used to living the domestic dimension. This reflection is supported by the literature (Carriero and Todesco, 2016) which highlights how Italian women are more dedicated to caring for the home, even during the pandemic period (Rania et al., 2020), while men are more used to carrying out activities outdoors. Although technologies, like Facebook and other social networks, have certainly contributed to making people feel more connected, contrasting the feelings of loneliness as highlighted by some recent works (Cho, 2015; Knausenberger and Echterhoff, 2018), the data showed that those who lived alone perceived greater loneliness than those who lived with partner or with partner and children. This can be explained by the fact that those who lived alone had a more intense perception of the lack of social contacts, caused by compulsion at home. In fact, as Tull et al. (2020) argue, the reduction of contacts due to the emergency leads to increased feelings of loneliness and social isolation.

The variables considered are all related to each other, highlighting that with the decrease in happiness, due to the situation experienced by people in this period, a worse perception of mental health increases. The GHQ, in fact, evaluates both the perception of anxiety and depression and the well-being and social functioning dimensions that were both put in crisis by the forced lockdown. Furthermore, there are also positive relationships between the perception of loneliness and mental malaise and negative relationships between loneliness and happiness as also found in the literature (Cacioppo et al., 2010; Lee et al., 2019; Chang et al., 2020).

In support of these results, the regression analyses highlighted the dimensions that influence the perception of mental health in the lockdown period. The lower level of happiness and the greater perceived loneliness as well as the size of the home had effects on the participants’ perception of mental health. In fact, Yang and Ma (2020) found that sharing a limited space for long periods of time could have had an impact on a couple’s relationship, and therefore on psychological well-being. On the other hand, regarding the construct of loneliness, the predictors are happiness, mental health, the persons with the subject lives, and education. Obviously living alone makes you perceive the condition of loneliness more in a period in which compulsion at home does not allow you to experience external relations. Indeed, Killgore et al. (2020) found that having to at stay home because of the pandemic has a negative impact on the perception of loneliness and social disconnection. Porcelli (2020) also found that loneliness combined with anxiety and fear is one of the most dangerous consequences of the condition of social isolation that has been forced on us for a very long period. The variable education also affects the level of loneliness. This figure is in line with the literature which highlights how low levels of education can lead to a significantly higher level of psychological symptoms (Tian et al., 2020).

These data must make researchers, policymakers, and psychologists working in the field reflect on the fact that the data were collected 2 weeks after the lockdown and for the next 10 days, and therefore it is presumable that with the passing of the lockdown, this perception of malaise could also be accentuated.

## Implications for Practice

These data should be brought to the attention of political decision-makers: although it is true that the medical health dimension remains a particularly relevant and essential aspect, it nevertheless seems appropriate to also take into account the dimension of psychological well-being of the population, which at this moment appears to be particularly strained. Considering that these data were collected in the first quarantine period, it would be appropriate to collect further data at a later date given the prolonged situation in order to track the trend of the psychological variables severely tested by the lockdown period and by the climate of medical and economic uncertainty being faced.

Therefore, it is important to monitor the progress of these psychological dimensions to intercept any alarm signal for both the population affected by COVID-19 as highlighted in the literature (Bo et al., 2020), both for the general population and to prevent long-term consequences.

We therefore believe that our data could be useful for hypothesizing future and diversified interventions to be applied in situations comparable to the current one: considering the psychological dimension of the subjects from the outset could allow participants to more effectively face mandatory lockdown at home and to feel more involved in political decisions and not to experience them as impositions. This is especially important since it is very likely that there may be a resurgence of this virus as has already happened in the past with similar viruses (Pathan et al., 2020). As highlighted by Yang et al. (2020), the lessons learned during the SARS epidemic and now during the SARS-CoV-2 pandemic may provide elements of reflection and help build a response capacity for future situations in order to be ready for future epidemics. Moreover, at the moment, there is no scientific evidence that people who have had COVID-19 have developed antibodies and are therefore protected from reinfection (World Health Organization, 2020b). Therefore, a possible resurgence of the virus cannot be excluded, also linked to the failure to reach herd immunity, as sustained in some countries.

This research has some limitations. In fact, it can be easily seen from the sociodemographic data that most of the participants have a high level of education, which is not very representative of the general population even if the questionnaire was distributed through various social networks accessible to all groups of the population. Therefore, another limit can be linked to the online administration of the tool used, which despite the researchers’ efforts could have influenced the involvement of some target populations. However, due to the contextual situation that involved forced physical distancing, the online questionnaire method seemed the only possible strategy to reach a large number of subjects and has already been used by other researchers in relation to the COVID-19 epidemic (Wang Y. et al., 2020). Finally, another limit is represented by the gender of the participants; in fact, although the data emerged regarding the gender difference are interesting, it should be emphasized that 75.5% of the participants are female.

Nonetheless, the data have revealed interesting aspects to consider in order to face with greater awareness critical situations and forced quarantine due to new waves of COVID-19 or future viruses (Yang et al., 2020). Despite the weaknesses highlighted, the study’s strengths include the fact that it is one of the first carried out on the lockdown period linked to the COVID-19 pandemic in Italy that aims to investigate the psychological dimensions of individual well-being.

Although the quarantine measures adopted in several countries have reduced deaths, the impacts of the COVID-19 pandemic are not limited only to the medical aspects, but its sociological, psychological, and economic effects at a global level will have repercussions not only in the immediate short term but also in the following months. Therefore, the longitudinal approach of our study is undoubtedly a strength that will allow us to monitor the well-being and mental health aspects of the general Italian population.

## Data Availability Statement

The raw data supporting the conclusions of this article will be made available by the authors, without undue reservation.

## Ethics Statement

The studies involving human participants were reviewed and approved by the Ethics Committee of the Department of Education Sciences of the University of Genoa. The participants provided their written informed consent to participate in this study.

## Author Contributions

NR conceived of the presented idea and supervised the findings of this work. NR and IC developed the theory, performed the quantitative analyses, and wrote the manuscript. Both authors discussed the results and contributed to the final manuscript.

## Conflict of Interest

The authors declare that the research was conducted in the absence of any commercial or financial relationships that could be construed as a potential conflict of interest.
